# Multicystic encephalomalacia: An autopsy report of 4 cases

**DOI:** 10.4322/acr.2020.208

**Published:** 2020-11-20

**Authors:** Manoj Gopal Madakshira, Kirti Gupta, Preithy Uthamalingam, Gargi Kapatia, Shiv Sajan Saini

**Affiliations:** 1 Post Graduate Institute of Medical Education and Research, Department of Histopathology, Chandigarh, India; 2 Royal Liver pool and Broadgreen University Hospital NHS Trust, UK

**Keywords:** Brain, Encephalomalacia, Hypoxia, Gliosis

## Abstract

Multicystic encephalomalacia is varying sized cystic lesions in the brain encountered in developing fetuses or infants. These cysts start at the periventricular area and may extend onto the cortex. The cause of the formation of these cystic lesions is secondary to an ischemic or hypoxic insult, which leads to liquefactive necrosis and subsequent formation of gliotic cyst walls having an admixture of microglia. We discuss four autopsy cases that had multicystic encephalomalacia to highlight the scenarios in which these lesions are encountered.

## INTRODUCTION

Multicystic encephalomalacia was recognized as a gross manifestation at autopsy in early infants by Wolf and Cowen in 1954 while studying a series of deaths related to cerebral atrophy and encephalomalacia.^1^ Multicystic encephalomalacia is characterized by varying sized cystic spaces, usually in the cerebral matter, without communication with the ventricular system.^2^ There is no genetic basis to this entity and is usually the result of prenatal or perinatal hypoxic insult to the brain.^3^ Other etiologies ascribed to the formation of multicystic encephalomalacia include viral encephalitides such as herpes simplex^4^ and cytomegalovirus,^5^ rare instances of lactic acidosis due to severe congenital metabolic disorders^3^ ^,^ ^6^ and secondary to abusive head trauma.^7^ This entity can be recognized by serial transcranial ultrasonography in susceptible infants as the change is irreversible and prompt management of the inciting cause reduces the disability in the infant.^8^ ^,^ ^9^ This entity is primarily described in the early literature. Herein, we discuss and illustrate 4 cases of multicystic encephalomalacia seen at autopsy and the clinical symptomatology leading to the death in these neonates and infants.

## AUTOPSY FINDINGS

### Case 1

**Figure 1:**
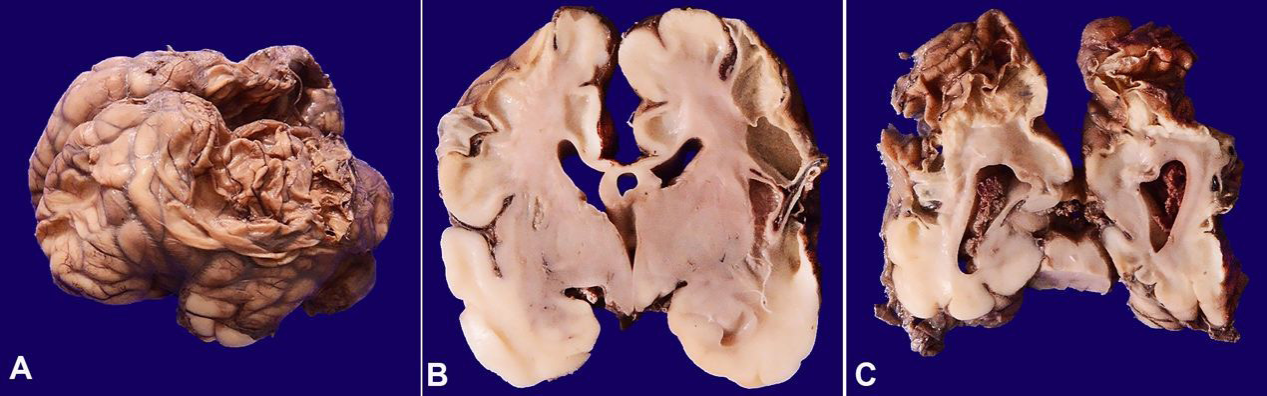
Gross findings of the central nervous system showing in **A –** Right lateral view of brain with a depression in the parietal region with collapse of the meninges, **B and C –** Coronal slices of brain with multiple cystic spaces in central white matter and extension onto the overlying cortex.

A 34-week-old (gestational age) premature male, with a small for gestational age, birth weight of 1221 g, (Expected weight 2100 g) was born following spontaneous onset of labor with preterm vaginal delivery. However, the baby did not have a spontaneous cry with an Apgar score of 1,1,0, and 0 (Scored at 1, 5 10 and 15 minutes after birth). Attempts at resuscitation could not revive the baby. The baby was a product of Gravida 5 with antenatally detected oligohydramnios secondary to renal agenesis and suspected congenital heart disease. The possible cause of the demise of the baby was clinically suspected to be due to antenatal hypoxemia or ischemia secondary to lung hypoplasia. On autopsy, the facies was normal. The brain weighed 210g (Reference range (RR) 280+ 3 g), and had a normal gyral pattern. There was a depression of 2cm in the right parietal region with the collapse of meninges (Figure 1A). Coronal slicing showed multiple cystic spaces in central white matter extending to the overlying cortex (Figure 11C), which on the microscopy revealed large areas of cystic degeneration lined by microglial cells and regions of gliosis. Clusters of paraventricular neurons were still evident. Amongst the extracranial organs, there was bilateral renal agenesis, bilateral hypoplastic lungs and liver revealed features of sinusoidal congestion and extramedullary hematopoiesis out of proportion to the gestational age. The heart was within normal limits and the placenta was not available for examination. Thereby, the case was diagnosed as cystic encephalomalacia with Potter’s sequence.

### Case 2

**Figure 2:**
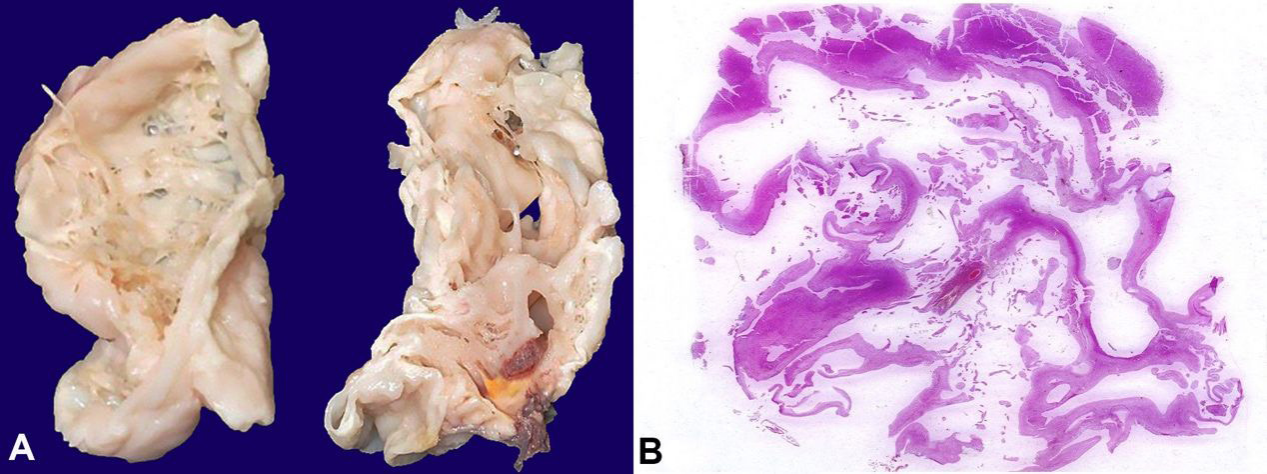
Gross findings of the central nervous system showing in **A –** Sagittal slices showing multiple cysts in central white matter with peripheral thinned out cortical ribbon, **B –** Photomicrograph of the whole mount section with cavitation involving the frontal lobes. (H&E, 1X).

A small for gestational age male baby with a birth weight of 1138 g (Expected weight 1700 g) was born at 32 weeks following spontaneous onset of labor by preterm vaginal delivery. The baby cried immediately after birth with an Apgar score of 8 and 9. On day 3 of life, the baby developed feed intolerance, metabolic acidosis, and thrombocytopenia. He was managed as a case of Stage 2 necrotizing enterocolitis. He had episodes of hypoglycemia secondary to hyperinsulinemia, with blood culture being positive for *E coli* on Day 7. The neonate further developed conjugated jaundice and anemia requiring blood transfusion. He rapidly deteriorated with worsening of abdominal distension and sclerema. Septic shock and capillary leak syndrome preceded the demise of the baby on day 8. In retrospect, the baby was a gravida 2 neonate with no major antenatal problems. The brain at autopsy weighed 179 g (RR; 238+2 g). The gyral pattern was normal. Externally, the meninges were shriveled and collapsed over the parieto-occipital region (5x5x2cm) bilaterally because of the loss of brain parenchyma underneath. Otherwise, the meninges were congested and dull. Coronal slicing revealed multiple cysts in central white matter with peripheral thinned out the cortical ribbon. The right side was more affected than left, and the involvement was more severe in the posterior segments compared to the frontal region (Figure 2A). The brain stem and rest of the cerebellum were essentially normal. The microscopy of affected regions was similar to the first case. However, the cystic change was more marked with cavitation, and involvement was seen in the frontal lobes bilaterally, left basal ganglia, right insular cortex, and bilateral parietal lobes (Figure 2B). Sections from the brain stem revealed features of early ischemia. The extracranial organs, including the liver, demonstrated sinusoidal congestion and lungs showed features of hyaline membrane disease. The intestines did not show any specific pathology and the placenta was not available for examination. Sepsis was attributed as the possible etiological factor resulting in encephalomalacia.

### Case 3

**Figure 3:**
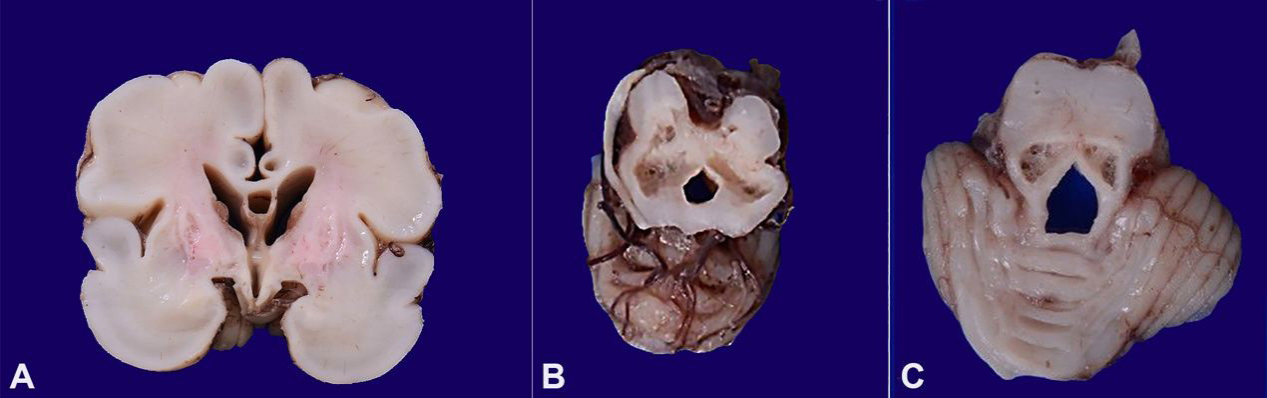
Gross findings of the central nervous system showing, in **A –** Coronal slices with softening the cystic change in the basal ganglia, **B and C –** axial slices with cystic change in the midbrain and tectum of pons.

A male baby who was appropriate for gestational age, with a birth weight of 1600 g (Expected weight 1700 g) was born at 32 weeks following emergency lower segment caesarean section indicated by poor maternal efforts and poor biophysical profile. The baby did not cry immediately after birth recording an Apgar score of 1,2, 2, and 2 (Scored at 1, 5, 10 and 15 minutes after birth), which demanded positive pressure ventilation for 90 minutes. The child developed profound encephalopathy, which was explained to the parents, who took an informed decision to withdraw life support. The mum was gravida 6 with a history of maternal diabetes, polyhydramnios, and poor biophysical profile. The brain at autopsy weighed 212 g (RR; 328+2g) with a normal gyral pattern. On coronal slicing, extensive softening and cystic change were noted in the cingulate gyrus, basal ganglia, and midbrain causing loss of central white matter (Figure 3AC).

**Figure 4:**
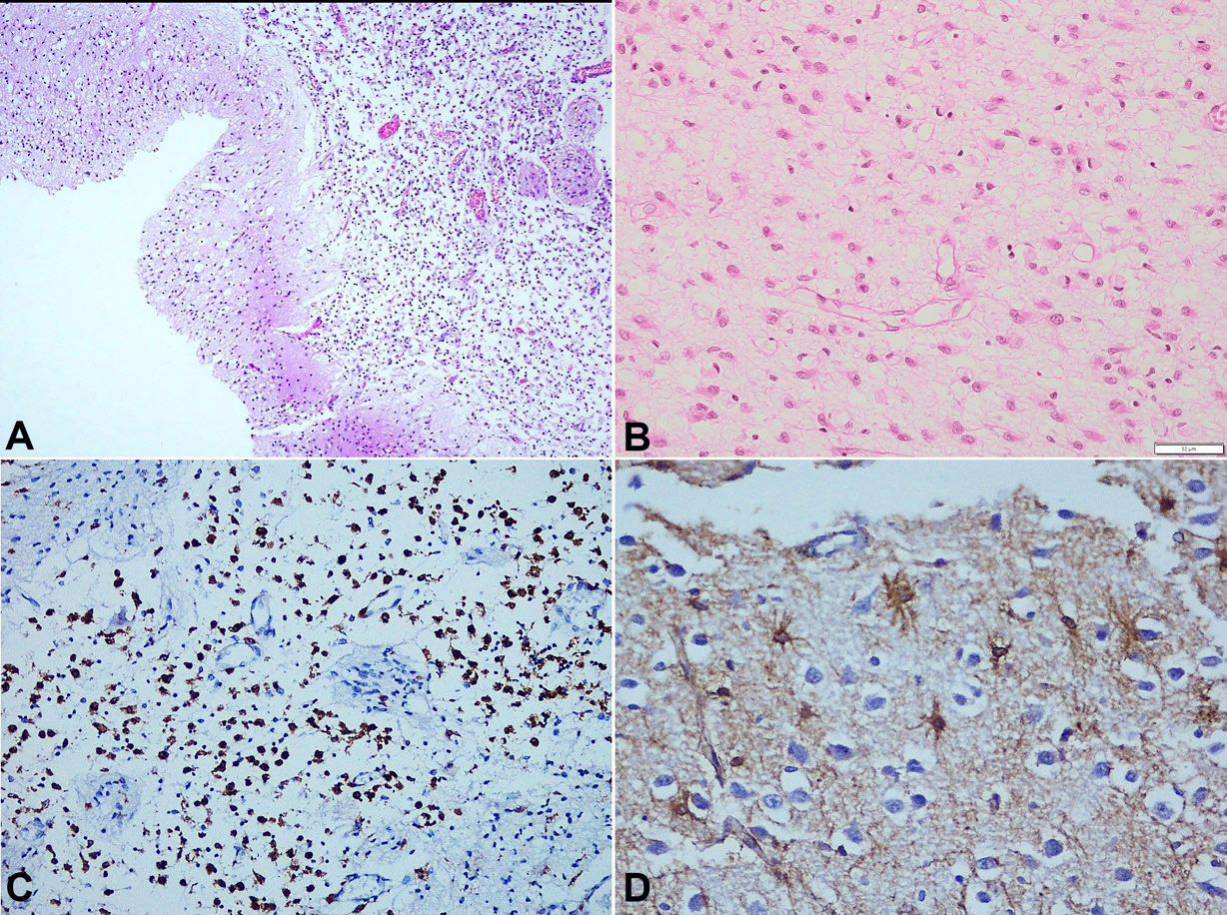
Photomicrograph of the Central nervous system with **A –** Sections from cyst wall lined by gliotic brain parenchyma with surrounding edema and microglia (H&E, 4X), **B –** Sections from cyst wall with increased density of gemistocytes having abundant dense eosinophilic cytoplasm (H&E, 10X), **C –** Sections from cyst wall highlighting the astrocytes (GFAP, 40X), **D –** Sections from cyst wall highlighting the microglia (CD68, 10X).

Secondary dilatation of lateral and third ventricle was noted. Similar cystic change was noted in the entire brain stem, including tectum of pons and medulla. Microscopically, the cystic spaces were bordered by microglial cells and gliosis with neuropil pallor and edema noted in the surrounding regions (Figure 4A). The gliotic areas showed prominent reactive gemistocytes highlighted by GFAP immunohistochemistry (Figure 44C). The microglial cells were highlighted by CD68 immunohistochemistry (Figure 4D). In the extracranial organs, bilateral pulmonary hypoplasia (Combined weight of 30 g, Lung /body weight of 1.8%, against normal of 2.25%) was noted. However, both kidneys were normal. The other organs were within normal limits and the placenta was not available for examination. Placental insufficiency was suspected to the possible etiology behind cystic encephalomalacia.

### Case 4

**Figure 5:**
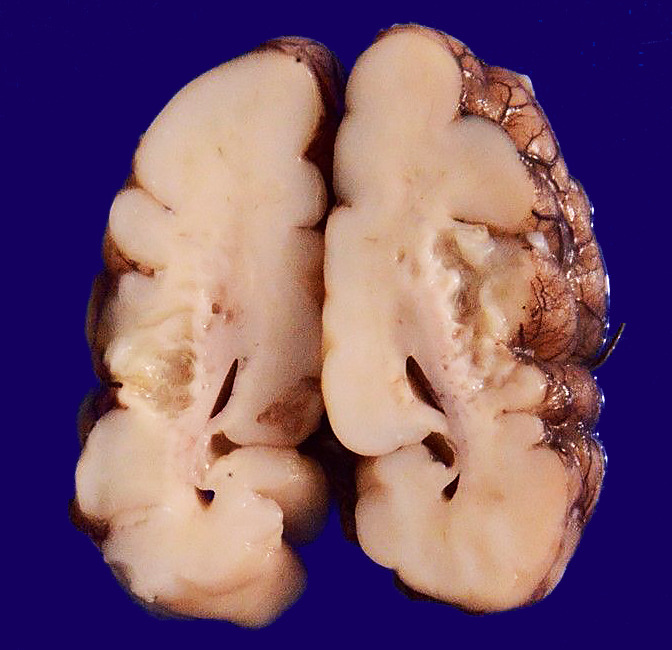
Gross findings of the central nervous system showing cystic cavities involving the grey and white matter.

A 2210 g birth weight (Expected weight 2100 g) appropriate for gestational age male baby, was delivered at 34 weeks of pregnancy by vaginal delivery. The child cried immediately at birth with an Apgar of 8 and 9 (Scored at 1 and 5 minutes after birth). The child was started on breastfeeding on day 3 of life. On Day 21, the newborn was readmitted with complaints of poor feeding, lethargy, and respiratory distress for 3 days. The parents also gave history suggestive of seizures on 1 day. On examination, the child had severe encephalopathy with sluggish pupillary reflexes. Ultrasonography showed grade 3 intraventricular hemorrhage with bilateral cystic periventricular leukomalacia. Blood cultures showed growth of Enterococcus and group A beta-hemolytic streptococci. The cerebrospinal fluid analysis was indicative of meningitis. The baby showed improvement with antibiotics and supportive care and was discharged on oral feeds. A few days later on day 34, the baby was brought in a state of cardiac arrest and could not be revived. At autopsy, the brain showed normal gyral pattern; however, the meninges were collapsed over parietal regions. On coronal slicing, there was the presence of multiple cystic cavities in the central white matter, also involving the grey matter with resultant communicating hydrocephalus (Figure 5).

The cystic spaces microscopically were lined by microglial cells and occasional lymphocytes with reactive gliosis. Mild inflammatory infiltrate with the subarachnoid space was noted indicating meningitis. Lungs revealed features of capillary hemangiomatosis, in the form of prominence of dilated capillaries in the alveolar septe, secondary to hypoxia. The remaining organs were normal.

## DISCUSSION

Multicystic encephalomalacia is a gross description defined by the presence of multiple cystic degenerative spaces, which commonly involve the cerebral grey matter and basal ganglia preferentially.^10^ The cystic spaces may have random distribution and have varying sizes depending on the type of insult and duration of the inciting damage.^11^ The cystic spaces may rarely involve the cerebral white matter and brain stem. Authors have used many terms to describe these cystic lesions such as pseudoporencephaly, encephalodystrophia, polyporencephaly, or multilocular encephalomyelomalacia.^2^ The most common hypothesized event causing multicystic encephalomalacia is a hypoxic insult in the third trimester or perinatal period.^10^ In contrast, insult before 24 weeks, at the time when cortical plate neurons are in the last phase of migration results in cytoarchitectural and gyral disorganization.^12^ The cause of cystic formation is due to the liquefactive necrosis of the infarcted area and occasionally involving regions of germinal matrix hemorrhage, following the hypoxic insult.^10^ The hypoxic insult can be due to reduced oxygen uptake, such as asphyxia or carbon-monoxide poisoning, disturbed systemic blood flow secondary to hypovolemic shock, or compression of vasculature.^7^ The hypoxic insult results in liquefactive necrosis of the susceptible grey matter areas which eventually leave behind the cystic spaces. However, the distribution of the cystic degeneration does not conform to any specific vascular network distribution. These observations and the rarity of the occurrence of cystic encephalomalacia in most hypoxic infants point towards additional unknown factors that precipitate or accrue towards its formation.^2^ The rare occurrence of cystic spaces in the white matter can be explained by the possible sequence of events. The initiating step remains hypoxia, which causes altered permeability of the blood-brain barrier causing cerebral edema, venous congestion, and tissue acidosis. The increased acidosis and worsening of the cerebral edema finally cause myelin breakdown and damage to the white matter.^13^ ^,^ ^14^ Some of the other factors associated with multicystic encephalomalacia include viral encephalitis. There have been many cases of herpes and cytomegalovirus encephalitis, which shown a cystic change in the brain. The postulated explanation for these changes is secondary to the cytotoxic damage by the virus and the infiltrating cytotoxic inflammatory cells on the brain parenchyma.^4^ ^,^ ^5^ Some reports have also shown the formation of these cystic lesions in cases of inborn errors of metabolism with severe congenital lactic acidosis. The hypothesized mechanism in such cases is the severe cytotoxic metabolic damage to the susceptible neural tissue, leading to cell death.^3^ ^,^ ^6^ Instances of multicystic encephalomalacia have also been reported in infants who have been victims of abusive head trauma. The postulated mechanism is the development of cerebral edema and compressive subdural hematoma following abusive head trauma, which result in secondary infliction of hypoxic injury to the brain parenchyma.^7^ Placental examination was not available in our series. However, placental examination may reveal the cause of the etiological factors leading to cystic encephalomalacia in the fetus, which include: placenta previa, abruptio, abnormalities in placental circulation and tell-tale signs of infections.^15^ ^-^ ^17^

The sequence of events leading to the formation of cysts begins with liquefactive necrosis of the brain parenchyma, which forms the acute phase lasting about 8 to 24 hours. This is followed by a subacute inflammatory phase of 3 to 5 days, which is characterized by macrophage infiltration, rimming by reactive astrocytes, and formation of axonal spheroids. The final phase it that of chronicity and repair, which is essentially a continuation of the subacute phase with the addition of formation of cystic cavities with accompanying ventriculomegaly.^18^ Multicystic encephalomalacia needs to be differentiated from hydranencephaly and porencephaly, by the presence of multiple cysts involving multiple lobes separated by glial septa.^18^ The early recognition of multicystic encephalomalacia is due to the irreversible damage to the brain, which manifests severe intellectual damage and intractable epilepsy, which is unresponsive to antiepileptics. The lesion usually progresses to spastic quadriparesis, dementia, and death.^10^ The screening modality of choice in susceptible infants is serial cranial ultrasonography. Neuro-sonography has the benefit of being non-invasive bed-side modality without the use of radiation and has been proven to reliably assess the size of the cystic lesions and the need for shunts.^8^ ^,^ ^9^ The rapidity of development of these lesions in the perinatal period, call for early and frequent screening to enable early diagnosis.
